# Coronary atherosclerosis on AI-based plaque analysis in patients with chest pain and calcium score zero

**DOI:** 10.1007/s10554-026-03623-x

**Published:** 2026-01-19

**Authors:** David Molnar, Juhani Knuuti, Jeroen J. Bax, Antti Saraste, Teemu Maaniitty

**Affiliations:** 1 Turku PET Centre, Turku University Hospital, University of Turku, Kiinamyllynkatu 4-8, Turku, 20520 Finland; 2 Department of Molecular and Clinical Medicine, Institute of Medicine, Sahlgrenska Academy, University of Gothenburg, Gothenburg, Sweden; 3 Department of Clinical Physiology, Nuclear Medicine and PET, Turku University Hospital, Turku, Finland; 4 Department of Cardiology, Leiden University Medical Center, Leiden, The Netherlands; 5 Heart Center, Turku University Hospital, University of Turku, Turku, Finland

**Keywords:** Non-obstructive coronary artery disease, Artificial intelligence-based quantitative computed tomography, Clinical reading, Sex-differences, Long-term outcome

## Abstract

There is conflicting evidence on the prevalence of obstructive and non-obstructive coronary artery disease (CAD) and outcome in symptomatic patients with zero coronary artery calcium score (CACS). To characterize sex differences in coronary atherosclerosis in patients with chest pain and zero CACS. Patients evaluated for suspected CAD at Turku University Hospital 2006–2016 with zero CACS were included. In addition to clinical reading, artificial intelligence-based quantitative analysis (AI-QCT) of coronary computed tomography angiography (CTA) images was performed. Long-term clinical outcome of myocardial infarction, unstable angina or death was analyzed. Out of 2,216 patients with CTA results, 606 had zero CACS (156 men and 450 women, median age 54 vs. 61). Around 5% of men and 2% of women with zero CACS had obstructive CAD, with comparable findings on clinical CTA and AI-QCT. Non-obstructive disease was found in higher proportion on AI-QCT (88.5% of men and 85.6% of women) than on clinical reading (16.7% of men and 11.6% of women). Non-calcified plaques dominated, but small calcifications were not uncommon. Low-density plaques increased with the severity of CAD. After a median follow-up of 7.3 years, only 3 men and 14 women experienced clinical events, yielding an annual event rate of 0.26% in men and 0.43% in women. In patients with suspected CAD and zero CACS obstructive CAD is detected in around 5% of men and 2% of women. Non-obstructive CAD is detected in a vast majority with AI-QCT. Despite this, long-term clinical outcome is generally very good.

**Graphical abstract:**

Central illustration:

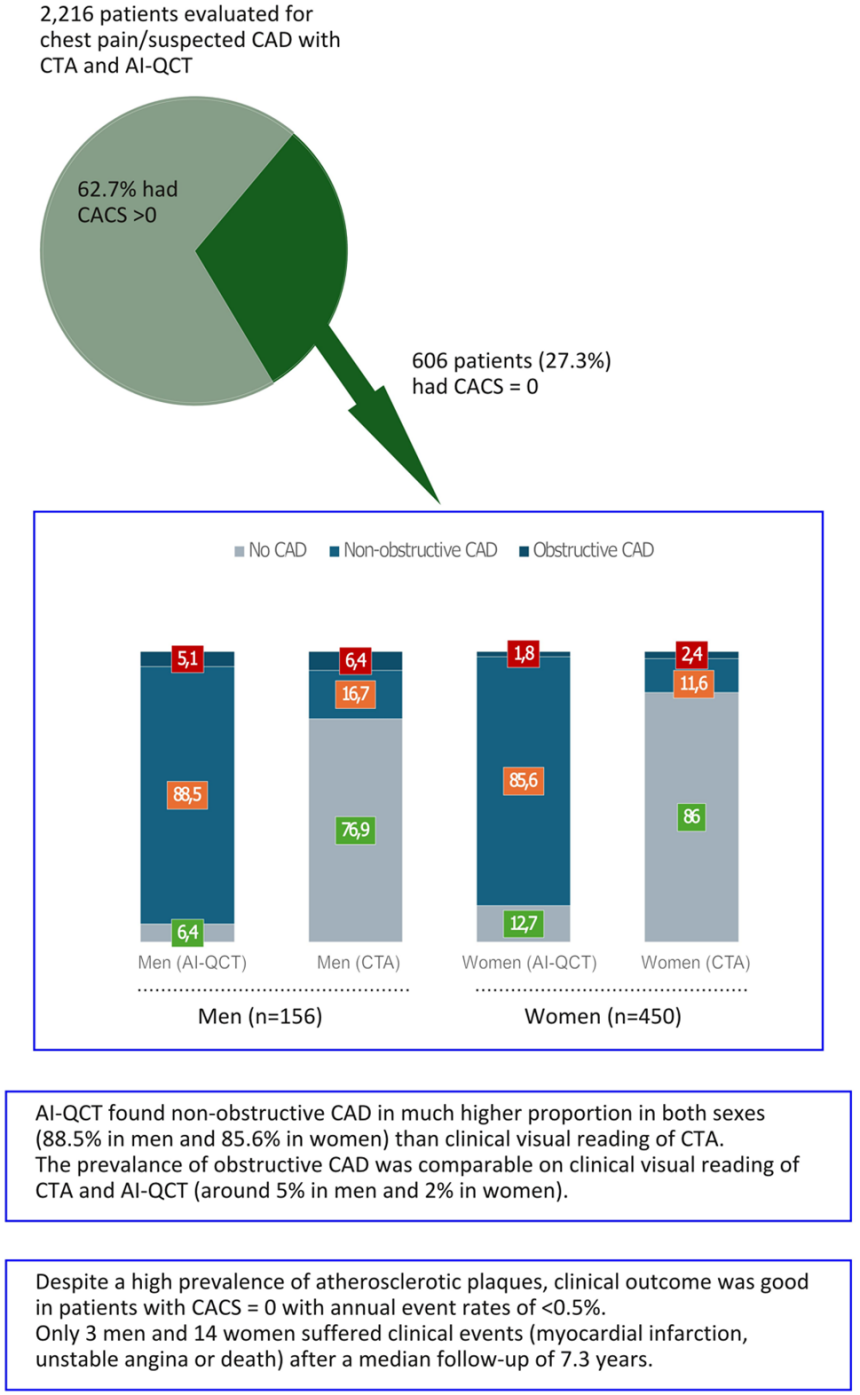

## Introduction

Computed tomography (CT) offers a convenient non-invasive method for in-vivo imaging of the coronary arteries. The coronary artery calcium score (CACS) [1], derived from non-contrast-enhanced CT, has since its inception been one of the most widely used and best described metrics for both the assessment of the extent of coronary atherosclerosis and risk prediction [2–4]. With the substantial technical evolution seen over the past decades, contrast enhanced imaging, coronary CT angiography (CTA), has been increasingly recognized to provide information beyond mere stenosis detection, specifically on plaque morphology and pathophysiological characteristics [5–7].

While calcification is acknowledged as one of the hallmarks of atherosclerosis, it is evident from numerous studies that a CACS of zero does not exclude coronary artery disease (CAD); there might even be severe, obstructive CAD in the absence of calcifications. In a meta-analysis including over 90,000 patients [8] the prognostic value of CACS of zero in patients presenting with chest pain was assessed and the authors concluded that the prevalence of obstructive CAD and annual risk of adverse events was very low, but not zero. Moreover, as the findings in a recent prospective study [9] of nearly 24,000 symptomatic patients imply, there seems to be an age differential in the utility of CACS of zero as a risk marker, with much lower reliability in younger age groups.

A series of high-risk plaque characteristics have been described on CTA [10–13], e.g., low-density plaques (LDP), spotty calcification, and positive remodelling. There is mounting evidence, that the prevalence of non-calcified plaques (NCP) might be higher than previously acknowledged. In a recent meta-analysis of asymptomatic individuals with CACS of zero covering work until March 2023 and including data from nearly 38,000 individuals [14], the authors pointed to a low prevalence of obstructive CAD (1.1%), but an overall prevalence of NCP of around 10%. Among symptomatic patients with chest pain who are found to have a CACS of zero, the prevalence of NCP is expected to be higher, but there is considerably less data available on plaque burden in this important subgroup.

Initially the morphological features of plaques were extracted by virtue of manual reading, which restricted the use in large scale population research due to time-consuming segmentation. At present, thanks to rapid developments in the field of computer vision, there are automatic or semi-automatic software solutions available for detailed plaque analysis, one of which is an FDA-approved, artificial intelligence-based algorithm for quantitative CTA analysis (AI-QCT) [15, 16].

In the present study, AI-QCT analysis was available from 2,216 patients evaluated for stable chest pain. The aim was to characterize the coronary plaque burden in patients having a CACS of zero with respect to: (a) the prevalence of obstructive CAD, (b) the prevalence of of different plaque components, and (c) sex and age differences related to a and b.

## Methods

### Study cohort

The study cohort was composed of symptomatic patients evaluated for suspected CAD between 2007 and 2016 at the Turku University Hospital in Finland. Patients with previously known obstructive CAD or prior myocardial revascularization were not considered for inclusion. Out of totally 2,411 consecutive patients, 195 (8.8%) were excluded due to non-retrievable CTA images or non-diagnostic image quality. Among the remaining 2,216 patients, who all had morphological plaque analysis performed with AI-QCT, a total of 606 patients had a CACS of zero and were included in the study (156 men and 450 women).

Demographic and clinical data were collected from electronic medical records. Long-term follow-up data on all-cause mortality, myocardial infarction and unstable angina pectoris was retrieved from hospital discharge registry data (Auria Clinical Informatics) and individually verified against electronic medical records.

### Informed consent and ethical permit

The study conforms to the Declaration of Helsinki and the study protocol has been approved by the Ethics Committee of the Hospital District of Southwest Finland without any requirement for written informed consent due to the observational study design.

### Computed tomography imaging

Non-contrast CT scans for CACS were first performed, followed by coronary CTA after the administration, as needed, of intravenous metoprolol (0–30 mg) to reach a target heart rate of 60 bpm, and isosorbide dinitrate aerosol (1.25 mg) or sublingual nitrate (0.8 mg) to dilate the coronary arteries. Low-osmolal iodine contrast was used. Two hybrid PET-CT scanners with 64-row detectors were used (GE Discovery VCT and GE D690, General Electric Medical Systems, Waukesha, USA). Preferably, prospective image acquisition was used. The protocol has been previously described in greater detail [17].

### Image analysis

CACS was calculated using the SmartScore 4.0 software (General Electric Medical Systems, Waukesha, USA) according to the procedures originally described by Agatston et al. [1]. Clinical analysis of CTA images was done on visual basis according to the recommendations of the Society of Cardiovascular Computed Tomography [18].

In addition, CTA images were re-analyzed in a blinded manner with an FDA-approved AI-QCT software (Cleerly LABS; Cleerly, Inc.; Denver, USA). It utilizes a series of convolutional neural networks to provide quantitative CTA metrics, which include the: degree of stenosis by diameter, total plaque volume (TPV), percent atheroma volume (PAV, or the TPV normalized to vessel volume), low-density plaque volume (LDP; ≤ 30 HU), non-calcified plaque volume (NCP; 31–350 HU), calcified plaque volume (CP; >350 HU). For all patients, CAD-RADS 2.0 classification [19] was derived from the AI-QCT analyses.

### Statistical analysis

All statistical analysis was performed in R [20] version 4.4.2 (2024-09-01) “Cranberry Hibiscus” release (a1fe401f, 2024-11-03) for windows. Statistical significance was defined as two-tailed *p* < 0.05. Test for normality was performed using visual examination of histograms and the Shapiro-Wilk test. Since continuous data was not normally distributed, the median with its interquartile range is reported throughout. Categorical data is reported as percentages. The Mann-Whitney U-test was used for assessing differences in continuous variables between groups, while the Fisher exact test was used to assess differences between groups for categorical variables. Linear regression analysis was performed for total plaque volume as an outcome of age, with both Spearman’s correlation coefficient and Kendall’s Tau calculated. Uni- and multivariable binary logistic regression analysis was performed as a sub-analysis for the presence of LDP, NCP and CP. All variables of potential interest were included in the univariable analysis, whereas the multivariable analysis was restricted to the variables being statistically significant in the univariable analysis: age and sex. All analyses except for the logistic regression modeling were performed with the data split for sex.

## Results

### Patient characteristics

There was an almost threefold preponderance of women in the selected cohort with a CACS of zero, 450 women (74%) and 156 men (26%). The median age of the women was higher by seven years (61 vs. 54 years) [Table 1]. The proportion of smokers was higher in men (36.5% vs. 20.7%). Other baseline characteristics were not significantly different between sexes, except for a slightly higher prevalence of typical angina in women (21.3% vs. 16.7% in men).

**Table 1:** Characteristics of the cohort and imaging results by sex and presence of significant stenosis (≥ 50% of vessel diameter). Continuous data is presented as the median with interquartile ranges in brackets and categorical data as percentage with number in parenthesis. Significant differences between sexes are noted with “A”, between stenosis groups within each sex with “B”, borderline significant with small letters

	Men (*n* = 156)		Women (*n* = 450)	
	All (*n* = 156)	Stenosis by AI QCT ≥ 50% (*n* = 8)	All (*n* = 450)	Stenosis by AI-QCT ≥ 50% (*n* = 8)
*Baseline characteristics*				
Age (years)	54^A^ [46.75–60.75]	56.5 [45.5–63.5]	61^A^ [53.25–66.25]	61 [57–65.5.5]
BMI	26.3 [24.8–29.3]	26.8 [25.2–28.6]	26.9 [24.2–30.9]	23.7 [23.1–25.8]
Smokers, current or previous [%]	36.5^A^ (*n* = 57)	62.5 (*n* = 5)	20.7^A^ (*n* = 93)	37.5 (*n* = 3)
Diabetes or pre-diabetes [%]	21.2 (*n* = 33)	37.5 (*n* = 3)	18.4 (*n* = 83)	25.0 (*n* = 2)
Pre-diabetes [%]	12.8 (*n* = 20)	37.5 (*n* = 3)	10.4 (*n* = 47)	12.5 (*n* = 1)
Hypertension [%]	33.3 (*n* = 52)	37.5 (*n* = 3)	40.9 (*n* = 184)	37.5 (*n* = 3)
Dyslipidemia [%]	52.6 (*n* = 82)	75.0 (*n* = 6)	53.3 (*n* = 240)	75.0 (*n* = 6)
Typical angina [%]	16.7^A^ (*n* = 26)	50.0 (*n* = 4)	21.3^A^ (*n* = 96)	25.0 (*n* = 2)
Atrial fibrillation or flutter, previous or present [%]	6.4 (*n* = 10)	0	8.0 (*n* = 36)	0
*AI-QCT results*				
CAD-RADS 0 [%]	6.4^A^ (*n* = 10)	0	12.7^A^ (*n* = 57)	0
CAD-RADS 1 [%]	80.8^A^ (*n* = 126)	0	78.4^A^ (*n* = 353)	0
CAD-RADS 2 [%]	7.7^A^ (*n* = 12)	0	7.1^A^ (*n* = 32)	0
CAD-RADS 3 [%]	0^A^ (*n* = 0)	0	0.9^A^ (*n* = 4)	50.0 (*n* = 4)
CAD-RADS ≥ 4 [%]	5.1^A^ (*n* = 8)	100.0 (*n* = 8)	0.9^A^ (*n* = 4)	50.0 (*n* = 4)
Presence of any plaque [%]	93.6^A^ (*n* = 146)	100.0^B^ (*n* = 8)	87.1^A^ (*n* = 393)	100.0^B^ (*n* = 8)
Presence of NCP [%]	93.6^A^ (*n* = 146)	100.0^B^ (*n* = 8)	87.1^A^ (*n* = 392)	100.0^B^ (*n* = 8)
Presence of LDP [%]	15.4^A^ (*n* = 24)	75.0^B^ (*n* = 6)	4.0^A^ (*n* = 18)	37.5^B^ (*n* = 3)
Presence of CP [%]	33.3^A^ (*n* = 52)	50.0 (*n* = 4)	43.3^A^ (*n* = 195)	37.5 (*n* = 3)
NCP volume [mm^3^]	30.5^A^ [15.4–58.6]	132.6^B^ [98.4–193.7.4.7]	16.7^A^ [6.6–34.4]	75.8^B^ [53.1–92.5]
LDP volume [mm^3^]	0^A^ [0–0]	3.1^B^ [0.6–12.2]	0^A^ [0–0]	0^B^ [0–0.4.4]
CP volume [mm^3^]	0^a^ [0–0.1.1]	0.05 [0–1.5.5]	0^a^ [0–0.6.6]	0 [0–2.2.2]
TPV volume [mm^3^]	31.1^A^ [15.48–59.83]	143.7^B^ [105.9–216.2.9.2]	18.1^A^ [6.8–35.5]	75.8^B^ [62.7–93.7]
*PAV [% of vessel volume]*				
PAV per patient, all vessels	0.9^A^ [0.5–1.6]	3.7^B^ [2.8–5.2]	0.7^A^ [0.3–1.3]	2.8^B^ [2.1–4.0.1.0]
PAV in LM	0 [0–3.8.8]	1.9 [0–4.5.5]	0 [0–2.2.2]	0.5 [0–2.3.3]
PAV in LAD	1.1^A^ [0.3–2.4]	5.5^B^ [4.5–11.0]	0.8^A^ [0–1.7.7]	4.9^B^ [3.6–7.2]
PAV in LCX	0.4 [0–1.3.3]	1.6 [0.4–3.0.4.0]	0.2 [0–1.0]	1.5^b^ [0.3–2.9]
PAV in RCA	0.5 [0–1.1.1]	1.4 [0.4–2.6]	0.4 [0–1.1.1]	1.4 [0.3–3.2]
*Stenosis ≥ 50% [% of pat.]*				
Any vessel	5.1^A^ (*n* = 8)	100.0 (*n* = 8)	1.8^A^ (*n* = 8)	100.0 (*n* = 8)
LM	0	0	0	0
LAD	4.5^A^ (*n* = 7)	87.5 (*n* = 7)	1.3^A^ (*n* = 6)	75.0 (*n* = 6)
LCX	0.6 (*n* = 1)	12.5 (*n* = 1)	0.2 (*n* = 1)	12.5 (*n* = 1)
RCA	0	0	0.4 (*n* = 2)	25.0 (*n* = 2)
*Stenosis 1–49% [% of pat.]*				
Any vessel	84.6 (*n* = 132)	N/A	78.4 (*n* = 353)	N/A
LM	26.3 (*n* = 41)	N/A	22.4 (*n* = 101)	N/A
LAD	66.7 (*n* = 104)	N/A	54.0 (*n* = 243)	N/A
LCX	46.2 (*n* = 72)	N/A	40.9 (*n* = 184)	N/A
RCA	55.1 (*n* = 86)	N/A	50.9 (*n* = 229)	N/A
Clinical CTA results				
*Obstructive disease:* *stenosis ≥ 50% [% of pat.]*				
Any vessel	6.4^A^ (*n* = 10)	100.0^B^ (*n* = 8)	2.4^A^ (*n* = 11)	87.5^B^ (*n* = 7)
LM	0	0	0	0
LAD	5.1^A^ (*n* = 8)	87.5^B^ (*n* = 7)	1.8^A^ (*n* = 8)	62.5^B^ (*n* = 5)
LCX	1.3 (*n* = 2)	25.0^B^ (*n* = 2)	0.4 (*n* = 2)	12.5^B^ (*n* = 1)
RCA	0.6 (*n* = 1)	0	0.4 (*n* = 2)	25.0^B^ (*n* = 2)
*Non-obstructive disease:* *stenosis < 50% [% of pat.]*				
Any vessel	16.7^A^ (*n* = 26)	N/A	11.6^A^ (*n* = 52)	N/A
LM	1.3 (*n* = 2)	N/A	1.1 (*n* = 5)	N/A
LAD	14.1^A^ (*n* = 22)	N/A	9.1^A^ (*n* = 41)	N/A
LCX	1.9 (*n* = 3)	N/A	1.1 (*n* = 5)	N/A
RCA	1.9 (*n* = 3)	N/A	2.0 (*n* = 9)	N/A

BMI = Body Mass Index, ECG = electrocardiography, CAD-RADS = coronary artery disease reporting and data system, NCP = non-calcified plaque, LDP = low-density plaque, CP = calcified plaque, TPV = total plaque volume, PAV = percent atheroma volume, LM = left main coronary artery, LAD = left anterior descending coronary artery, LCX = left circumflex coronary artery, RCA = right coronary artery

*Performed in 120 men and 300 women, **Performed in 83 men and 196 women

### Imaging results

Despite the fact that all included patients had a CACS of zero [Table 1], 6.4% of the men and 2.4% of the women had at least one obstructive coronary stenosis reported (≥ 50% of the vessel diameter) on clinical visual CTA reading. Non-obstructive CAD was reported in 16.7% of the men and 11.6% of the women, whereas the majority of patients (the remaining 76.9% of the men and 86% of the women) were classified as having no atherosclerosis on visual reading.

AI-QCT identified obstructive stenosis (≥ 50% of the vessel diameter) in 5.1% of the men and 1.8% of the women (CAD-RADS 3 or higher), which was in line with clinical reporting. However, with AI-QCT analysis, 88.5% of the men and 85.6% of the women had detectable non-obstructive atherosclerotic plaques, i.e., a total plaque volume of > 0 mm^3^ (CAD-RADS 1–2), and only 6.4% of the men and 12.7% of the women had no detectable coronary atherosclerosis (CAD-RADS 0) [Table 1].

**Fig. 1:**
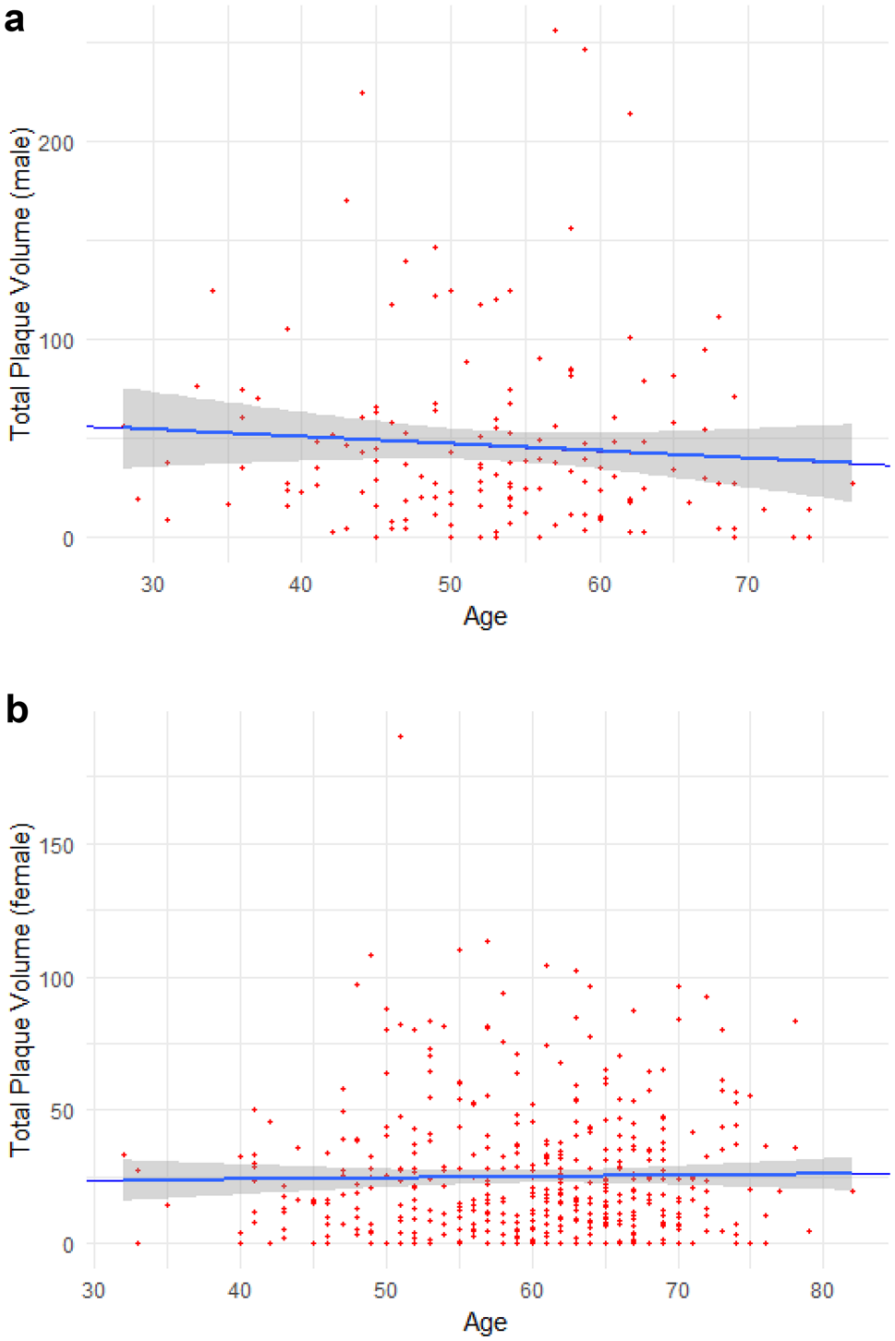
(**a**) Total plaque volume in mm^3^ as a function of age in men. Estimate: −0.36, SE = 0.38, *p* = 0.35. (**b**) Total plaque volume in mm^3^ as a function of age in women. Estimate: 0.049, SE = 0.13, *p* = 0.71

**Fig. 2:**
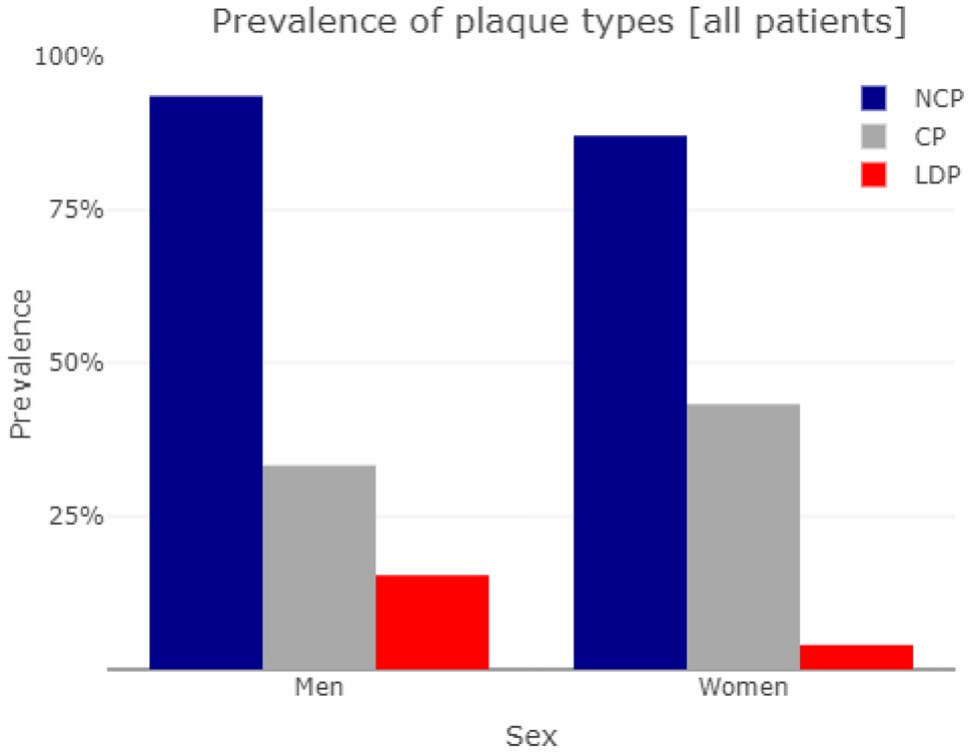
Prevalence of various plaque types in men and women on AI-QCT analysis of the coronary arteries. Non-calcified plaques (NCP), low-density plaques (LDP) and calcified plaques (CP) are shown

**Fig. 3:**
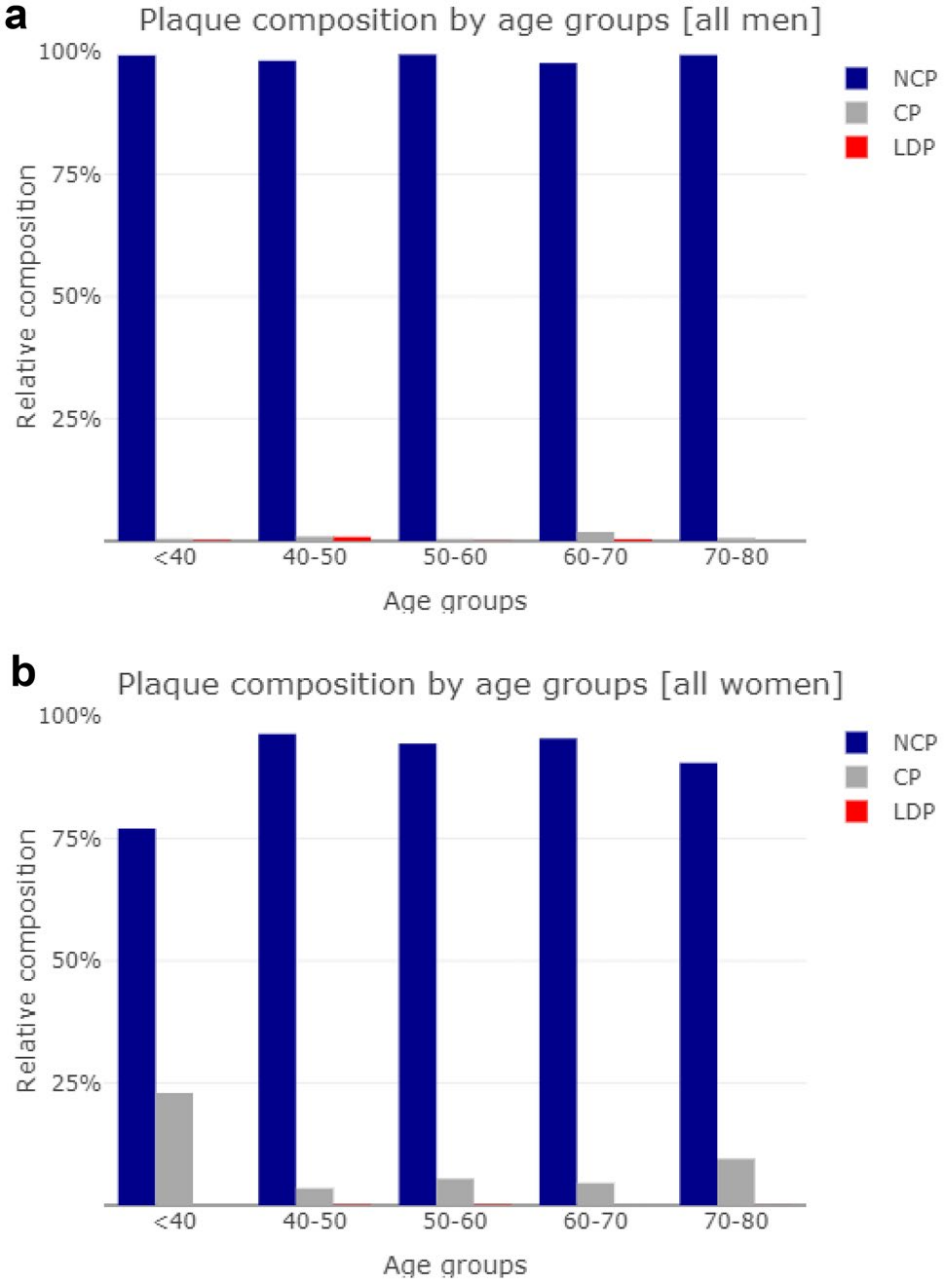
**a**. Relative composition of plaques in men by plaque types and age groups. Non-calcified plaques (NCP), low-density plaques (LDP) and calcified plaques (CP) are shown. **b**. Relative composition of plaques in women by plaque types and age groups. Non-calcified plaques (NCP), low-density plaques (LDP) and calcified plaques (CP) are shown

**Fig. 4:**
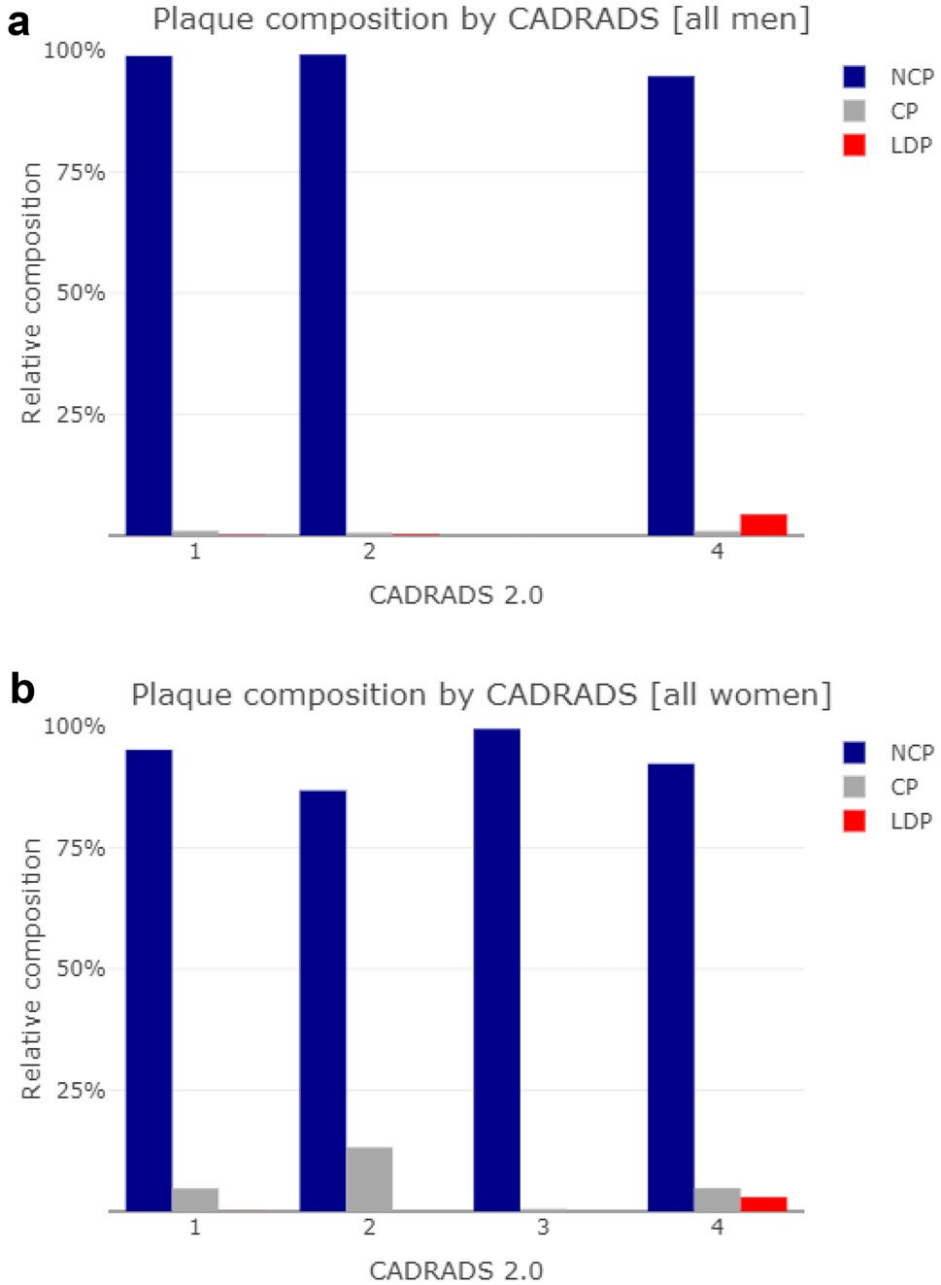
**a**. Relative composition of plaques in men by plaque types and severity of coronary atherosclerosis according to CAD-RADS 2.0 classification. Non-calcified plaques (NCP), low-density plaques (LDP) and calcified plaques (CP) are shown. No men were classified as CAD-RADS 3. **b**. Relative composition of plaques in women by plaque types and severity of coronary atherosclerosis according to CAD-RADS 2.0 classification. Non-calcified plaques (NCP), low-density plaques (LDP) and calcified plaques (CP) are shown

The total plaque volume showed a consistent distribution over age [Fig. 1a and b] in both sexes with a nearly horizontal linear regression slope (−0.36 in men, 0.049 in women, NS for both). Kendall’s tau showed similar results (−0.075 in men and 0.025 in women, NS for both). NCP was found in 93.6% of the men and 87.1% of the women, in both sexes accounting for the by far largest share of total plaque volume [Fig. 2]. The volumes of LDP and CP were small, but somewhat surprisingly CP were identified in 33.3% of the men and 43.3% of the women. The relative composition of plaques was not dependent on age [Fig. 3a and b], but showed an increasing proportion of LDP with increasing CAD-RADS group, more prominently in men [Fig. 4a and b], whereas the proportion of CP was higher in women, with a slight incline towards lower CAD-RADS group. By all metrics, the LAD was the most involved vessel in both sexes, men having both a higher burden of plaques (PAV of 1.1% vs. 0.8% in women) and more frequent occurrence of obstructive stenosis.

Considerable disagreement was observed between visual clinical CTA reading and AI-QCT among cases visually determined to have “no CAD” and “non-obstructive CAD”, corresponding to CAD-RADS 0–2 **[**Table 2**]**. Notably, in cases classified as “no CAD”, essentially compliant with CAD-RADS 0, few cases were assessed to have 0 TPV by AI-QCT, and by quartiles of TPV, the lower three (i.e. 75% of the 505 cases) had TPV of less than 50 mm^3^. Among cases found to have “non-obstructive CAD” on visual reading, i.e., compliant with CAD-RADS 1–2, the upper three quartiles (i.e. 75% of the 80 cases) had TPV of 15–247 mm^3^, while the lowest quartile (by TPV) included some cases with TPV of 0.

**Table 2:** Total plaque volume by its quartiles in visual clinical CTA reading ranging from “no CAD” to “non-obstructive CAD”

Visual clinical CTA reading	TPV Quartile	Median TPV	IQR	Range
No CAD (*n* = 505)	Q1 (0–25%)	0.4	0.00–4.20.00.20	0.00–6.80.00.80
	Q2 (25–50%)	12.3	9.33–15.88	6.80–19.20
	Q3 (50–75%)	27.1	23.15–31.45	19.20–36.30
	Q4 (75–100%)	53.35	42.90–67.55.90.55	36.30–155.50.30.50
Non-obstructive CAD (*n* = 80)	Q1 (0–25%)	7.2	0.00–9.97.00.97	0.00–15.10.00.10
	Q2 (25–50%)	22.9	17.88–24.95	15.20–28.00
	Q3 (50–75%)	37.25	33.77–43.85	29.40–54.30
	Q4 (75–100%)	82.5	77.93–98.00.93.00	55.60–246.50.60.50

### Predictors of plaque presence

In univariable logistic regression analysis sex, age, BMI, smoking, diabetes, hypertension, dyslipidemia, typical angina and atrial fibrillation/flutter were included for the binary outcome of presence or absence of NCP, LDP and CP. For NCP only sex was statistically significant (*p* = 0.03), whereas both sex and age were significant for LDP (*p* < 0.001, *p* = 0.04) and CP (*p* = 0.03, *p* = 0.003) [Table 3].

**Table 3:** Univariable logistic regression analysis with plaque components as binary outcome and cardiac risk factors as independent variables

Outcome	Variable	Coefficient	OR	CI 95	*P* value	Significance
NCP	Sex (female)	−0.7702	0.463	0.218–0.891	0.0304	*
	Age	−0.0102	0.99	0.963–1.016	0.458	NS
	BMI	0.0183	1.018	0.946–1.106	0.6441	NS
	Smoking	0.1686	1.184	0.661–2.238	0.5852	NS
	Diabetes	0.1539	1.166	0.981–1.370	0.0691	NS
	Hypertension	−0.0489	0.952	0.656–1.360	0.7919	NS
	Dyslipidemia	0.1239	1.132	0.761–1.675	0.5371	NS
	Angina	0.0796	1.083	0.923–1.275	0.3327	NS
	Atrial fibrill.	0.0538	1.055	0.810–1.370	0.6873	NS
LDP	Sex (female)	−1.4733	0.229	0.119–0.433	< 0.0001	***
	Age	−0.0335	0.967	0.937–0.998	0.0383	*
	BMI	0.0031	1.003	0.926–1.079	0.9358	NS
	Smoking	0.4533	1.574	0.784–3.027	0.1848	NS
	Diabetes	0.0514	1.053	0.839–1.382	0.6828	NS
	Hypertension	−0.2291	0.795	0.518–1.237	0.2996	NS
	Dyslipidemia	−0.1582	0.854	0.525–1.398	0.5248	NS
	Angina	−0.0857	0.918	0.748–1.119	0.4021	NS
	Atrial fibrill.	0.1781	1.195	0.858–1.696	0.3018	NS
CP	Sex (female)	0.4249	1.529	1.048–2.251	0.0291	*
	Age	0.0266	1.027	1.009–1.045	0.0029	**
	BMI	−0.0022	0.998	0.953–1.044	0.9225	NS
	Smoking	−0.1531	0.858	0.585–1.250	0.4281	NS
	Diabetes	0.0054	1.005	0.892–1.136	0.9304	NS
	Hypertension	−0.1193	0.887	0.705–1.118	0.3107	NS
	Dyslipidemia	−0.0329	0.968	0.750–1.248	0.7998	NS
	Angina	−1,00E-04	1	0.903–1.107	0.9979	NS
	Atrial fibrill.	−0.0665	0.936	0.790–1.108	0.4403	NS

In multivariable analysis, where age and sex were included for the binary outcome of presence or absence of NCP, LDP and CP, only sex retained significance for NCP (*p* = 0.04) and LDP (*p* < 0.001), while only age retained significance for CP (*p* = 0.01) [Table 4]. The changes in (natural) logarithmic odds ratio were − 0.76 and − 1.40 for female sex for NCP and LDP respectively, translating into: a 2.1 times greater odds of having NCP and a 4.05 times greater odds of having LDP with male sex. The OR for CP by age was 1.023, translating into a 2.3% increase in the likelihood of having CP for every year of age.

**Table 4:** Multivariable logistic regression analysis with plaque components as binary outcome and sex and age as independent variables

Outcome	Variable	Coefficient	OR	CI 95	*P* value	Significance
NCP	Sex (female)	−0.762	0.467	0.215–0.922	0.0383	*
	Age	−0.0013	0.999	0.971–1.027	0.9302	NS
LDP	Sex (female)	−1.3991	0.247	0.124–0.484	1,00E-04	***
	Age	−0.0114	0.989	0.956–1.023	0.5062	NS
CP	Sex (female)	0.281	1.324	0.891–1.982	0.1678	NS
	Age	0.023	1.023	1.005–1.042	0.0139	*

### Long-term clinical outcome

Data on clinical outcome was complete with the exception of 1 patient lost to follow-up. Only 3 men (1.9%) and 14 women (3.1%) had experienced adverse clinical events (myocardial infarction, unstable angina pectoris or death) during follow-up, the median duration of which was 7.3 years. These findings translate into an annual event rate of 0.26% in men and 0.43% in women. Due to the very low event rate the statistical power was insufficient to assess the impact of various risk factors and plaque components on outcome.

## Discussion

CACS of zero was present in 25% of the patients in our clinical cohort of symptomatic patients referred to coronary CTA due to suspected CAD. Detailed AI-QCT analysis of the coronary arteries yielded the following main findings in patients with CACS of zero: (1) obstructive coronary plaques are rare, but exist in about 5% of men and 2% of women; (2) non-obstructive plaques were detected in as many as 90% of men and 80% of women; (3) as expected, the plaques were mostly non-calcified, but around 30% of men and 40% of women had a some calcified plaque components; (4) the clinical outcome of patients with CACS of zero is generally very favorable, with annual event rates of below 0.5%.

Clinical visual reading and AI-QCT analysis showed similar findings with regards to the prevalence of obstructive coronary stenoses (≥ 50% of vessel diameter), whereas there was substantial discrepancy in the realms of subtle atherosclerosis detection. AI-QCT detected non-obstructive atherosclerosis (≤ 50% of vessel diameter) in the vast majority of the patients with CACS of zero, while clinical visual reading identified non-obstructive atherosclerosis in only around 17% of men and 12% of women. Unsurprisingly, the LAD was the most frequently affected vessel in both sexes by all metrics. The percent atheroma volume (PAV), which offers a normalized measure of plaque volume, was for instance around twice as high as for the LCX and RCA in both sexes.

The prevalence of both obstructive CAD and non-obstructive CAD on clinical visual reading in our study was generally in line with the findings of a large meta-analysis, which reported 3% prevalence of obstructive CAD and 13% prevalence of non-obstructive CAD in patients with stable chest pain and CACS of zero [8]. In contrast, AI-QCT analysis revealed coronary atherosclerosis in a vast majority of our cohort, raising questions whether or not these findings represent true, small atherosclerotic lesions that are overlooked on visual reading. A previous study by Choi et al. comparing AI-QCT findings to the consensus of three level 3 expert readers demonstrated a slight tendency towards higher plaque detection rate by AI-QCT in the lowest CAD-RADS categories [15], while Cardoso et al. found that 87% of small (0.1–50.0 mm3) plaques detected by AI-QCT remain in the same location on repeated CTA scans after an average follow-up of 3.8 years with an approximate three-fold increase in plaque volumes [21]. Finally, Omori et al. reported a strong corrrelation between plaque volumes on AI-QCT and invasive intravascular ultrasound with near-infrared spectroscopy, and proposed the use of non-zero cut-off values to improve the specificity of AI-QCT [22]. All of these studies suggest that AI-QCT findings do represent true atherosclerotic lesions and that AI-QCT seems to have an increased sensitivity for the detection of small lesions likely to be overlooked in clinical CTA evaluation. In our material, discrepancies between visual clinical CTA reading and AI-QCT were marked in cases corresponding to CAD-RADS 0–2, i.e., reflecting no or non-obstructive CAD. The overlap between visually “no CAD” and “non-obstructive CAD” is considerable, with AI-QCT as a reference, or inversely, if visual recordings would be the gold standard instead, AI-QCT would be unreliable in the classification of small plaques. While the granularity of the available clinical CTA data does not allow for any settlement of this intriguing question, as clinically active physicians in the field, we are inclined to believe that visual readings are less reliable with regards to small lesions of (hitherto) negligible consequences for the patients, since the presence or absence of obstructive CAD has been guiding further work-up and treatment. Notably, clinical reading, at least in our material, also appears to show a slight tendency to underestimate plaque burden more often in women relative to AI-QCT.o

In a recent work, Hollenberg et al., using another semi-quantitative analytical tool, likewise found high prevalence of non-obstructive stenosis (conforming to CAD-RADS 1 and 2) in patients with CACS of zero (40.1% progressing to 57.8% on follow-up up to 4 years later) although their number of patients was more limited [23].

Non-calcified plaques were the most common finding, with 94% of the men and 87% of the women having some. In terms of plaque volume, NCP was the dominating component, both LDP and CP being present in only minuscule volumes in most patients, although LDP was present in 15.4% of the men and 4.0% of the women, exhibiting the most significant sex difference in our material. In the group with CACS of zero, Hollenberg et al. also found an overwhelming dominance of NCP in the composition of plaques (97.9% decreasing to 91.9% on follow-up) [23], however their overall share of patients with any plaque detected was lower (< 60%). It is conceivable that the difference in NCP can be in part due to our cohort having a higher overall disease burden, while methodological differences, which are difficult to explore, could also contribute.

In our study, CP was present in minute amounts in 33% of the men and 43% of the women, despite CACS being zero. This discrepancy could largely be explained by lower sensitivity for small-volume calcifications in CACS analysis, using non-contrast images with a slice thickness of nearly five times that of CTA images. Artifacts from intravascular contrast might contribute, however, equally between sexes, thus not explaining our findings.

Somewhat unexpectedly, total plaque volume showed no significant relation to age on linear regression in either sex in our population with CACS zero. In patients belonging to CAD-RADS 3 or higher, i.e., having obstructive coronary stenosis of varying degree, there was a clear tendency towards increasing proportions of LDP in both sexes, consistent with the notion that LDP could be considered a high-risk plaque type [24]. CP had a more even distribution over CAD-RADS groups, and showed a weak association with age in logistic regression analysis independently of sex in our selected population. If non-calcified plaques are regarded as evolving entities, ultimately becoming calcified and stable, and plausibly in their end-stage, might lose some of their volume, these findings would make sense.

The women in our cohort were seven years older than the men (61 versus 54 years), and were nearly three times as many. In the large prospective study by Mortensen et al. [9] which included nearly 24,000 individuals, almost 13,000 had a CACS of zero, and the median age among them was 54 and 52 years for individuals with and without obstructive CAD. Notably, Mortensen et al. also had a higher proportion of women with CACS of zero, 7984 versus 4784 men, although in our material the skew is even more pronounced. The higher age among women is likely a function of delayed onset of atherosclerosis in women to past menopause [25], while the relative oversampling of women might stem from a lower CAD burden in women with chest pain, with an increased pre-test probability to have a CACS of zero.

Since the clinical outcome was very good, with annual event rates of below 0.5%, statistical analyses on factors contributing to events yielded no meaningful results, despite the fact that we had high-quality clinical data and a long follow-up. Further studies in larger cohorts and possibly with even longer follow-up are needed to better understand the potential prognostic significance of subtle atherosclerosis detected by AI-QCT.

### Strengths and limitations of the study

The main strength of the present study is the high quality imaging data, based on both standard clinical CACS and CTA reading and AI-QCT analysis, available together with long-term follow-up data from a real-world clinical cohort. Although AI-QCT has previously been extensively documented and tested and compares well to expert visual reading, some questions remain with regards to the generalizability of these studies. Automatic measurements seemingly increase the sensitivity for detection of small lesions, but the clinical importance of this is not clear from our results, and need to be further explored. Also, AI-QCT detects more calcifications as compared to CACS, likely due to both thinner slices with higher resolution in the z-plane, and potential artifacts from intravascular contrast. In light of this, the clinical significance of CP volumes on AI-QCT remains unclear. On the positive side, AI-QCT is expected to be less influenced by random interpretative errors and bias, which could arguably be an issue in a clinical setting, where readers are not blinded to factors that could influence decision-making, e.g., patient history and sex.

The main weakness of the present study would unequivocally be the relatively small size of the cohort, and the inherently skewed distribution of sex towards a female majority by almost three times. This fact makes reduces the potential for generalizability of some of the results. The relatively small number of patients with obstructive coronary stenosis (eight men and eight women) and very favorable clinical outcome limited statistical power.

## Conclusion

In symptomatic patients with CACS of zero obstructive coronary plaques are found in around 5% of men and 2% of women. However, a vast majority of patients have non-obstructive atherosclerosis detected by AI-QCT, which has a higher likelihood of detecting non-obstructive CAD than clinical visual reading. Plaques are mostly non-calcified, but a relatively higher proportion of low-density plaques are present in obstructive CAD, and calcified plaques are present only in very small volumes. The long-term clinical outcome in individuals with CACS of zero is generally very good.

## Data Availability

Data not provided in the manuscript can be provided upon reasonable request.

## Abbreviations

CAD: coronary artery disease
CACS: coronary artery calcium score
AI-QCT: artificial intelligence-based quantitative computed tomoghraphy
CTA: computed tomography angiography
LDP: low-density plaque
NCP: non-calcified plaque
TPV: total plaque volume
PAV: percent atheroma volume
CAD-RADS: coronary artery disease reporting and data system
CP: calcified plaque
LAD: left anterior descending coronary artery
LCX: left circumflex coronary artery
RCA: right coronary artery
